# Metabolomics of Esophageal Squamous Cell Carcinoma Tissues: Potential Biomarkers for Diagnosis and Promising Targets for Therapy

**DOI:** 10.1155/2022/7819235

**Published:** 2022-06-23

**Authors:** Jia Xu, Weiping Cao, Aizhong Shao, Ming Yang, Vivian Andoh, Qi Ge, Hui-wen Pan, Ke-ping Chen

**Affiliations:** ^1^School of Life Sciences, Jiangsu University, Zhenjiang, China; ^2^The Fourth People's Hospital of Zhenjiang, Zhenjiang, Jiangsu 212001, China; ^3^Department of Cardiothorac Surgery, Affiliated People's Hospital of Jiangsu University, Zhenjiang, China; ^4^School of Food and Biological Engineering, Jiangsu University, Zhenjiang, China; ^5^School of the Environment and Safety Engineering, Jiangsu University, Zhenjiang, Jiangsu 212013, China

## Abstract

**Background:**

The incidence of esophageal squamous cell carcinoma in China ranks first in the world. The early diagnosis technology is underdeveloped, and the prognosis is poor, which seriously threatens the quality of life of the Chinese people. Epidemiological findings are related to factors such as diet, living habits, and age. The specific mechanism is not clear yet. Metabolomics is a kind of omics that simultaneously and quantitatively analyzes the comprehensive profile of metabolites in living systems. It has unique advantages in the study of the diagnosis and pathogenesis of tumor-related diseases, especially in the search for biomarkers. Therefore, it is desirable to perform metabolic profiling analysis of cancer tissues through metabolomics to find potential biomarkers for the diagnosis and treatment of esophageal squamous cell carcinoma.

**Methods:**

HPLC-TOF-MS/MS technology and Illumina Hiseq Xten Sequencing was used for the analysis of 210 pairs of matched esophageal squamous cell carcinoma tissues and normal tissues in Zhenjiang City, Jiangsu Province, a high-incidence area of esophageal cancer in China. Bioinformatics analysis was also performed.

**Results:**

Through metabolomic and transcriptomic analysis, this study found that a total of 269 differential metabolites were obtained in esophageal squamous cell carcinoma and normal tissues, and 48 differential metabolic pathways were obtained through KEGG enrichment analysis. After further screening and identification, 12 metabolites with potential biomarkers to differentiate esophageal squamous cell carcinoma from normal tissues were obtained.

**Conclusions:**

From the metabolomic data, 4 unknown compounds were found to be abnormally expressed in esophageal squamous cell carcinoma for the first time, such as 9,10-epoxy-12,15-octadecadienoate; 3 metabolites were found in multiple abnormal expression in another tumor, but upregulation or downregulation was found for the first time in esophageal cancer, such as oleoyl glycine; at the same time, it was further confirmed that five metabolites were abnormally expressed in esophageal squamous cell carcinoma, which was similar to the results of other studies, such as PE.

## 1. Introduction

Esophageal cancer (EC) is a malignant tumor originating from the esophagus, which can occur in any segment of the esophagus and originates from esophageal epithelial cells. It is the eighth most common cancer in the world. Early symptoms are not obvious, often manifested as dysphagia, lack of early diagnostic biomarkers, the diagnosis is often advanced, and it is difficult to cure; globally, the overall five-year survival rate of patients with esophageal cancer is as low as 15% to 20%, indicating that it is associated with a poor prognosis [1, 2]. EC mainly includes two subtypes, esophageal adenocarcinoma (EAC) and esophageal squamous cell carcinoma (ESCC), which account for more than 95% of esophageal malignancies. Among them, squamous cell carcinoma mainly occurs in Asian and African countries, accounting for 90% of global cases. It is known as a “disease of developing countries,” and China has the highest incidence [3, 4]. Esophageal adenocarcinoma is the predominant type of esophageal cancer in North America and Europe, with a fivefold higher risk in whites compared with blacks [5, 6]. Therefore, esophageal cancer has more obvious geographical distribution characteristics than other common malignant tumors. According to the GLOBOCAN2020 database statistics (http://gco.iarc.fr/today), there will be 324,000 new cases of esophageal cancer in China in 2020, and the number of deaths will also be as high as 301,000, accounting for about 50% of the world and 60% of developing countries, with obvious local high incidence characteristics, especially in North China, forming typical epidemiological characteristics (Table 1) [7]. This also indicates that the incidence of esophageal cancer is closely related to factors such as geographical environment, and it is urgent to find and develop common and region-specific markers with the clinical application for the detection and treatment of esophageal cancer.

Metabolomics is the omics of simultaneous quantitative analysis of comprehensive profiles of metabolites in living systems, mainly using nuclear magnetic resonance (NMR) spectroscopy and mass spectrometry (MS), two common analytical techniques, for the analysis of findings in urine, plasma, or tissue. Characterization of small molecules, especially tissue analysis (especially valuable in the context of heterogeneous tissues such as the brain and cancer), can yield unambiguous biochemical information about disease mechanisms and thus provide new insights into disease-affected cell signaling pathways, generating new diagnostic biomarkers and new therapeutic approaches, maybe the most powerful way to study local and specific stimulus responses and pathogenesis today, and is now widely used in biomedicine, drug research, and other fields [8–12]. It is generally known that a distinctive feature in the development of malignant tumors is the alteration of metabolic processes and metabolic reprogramming, which is considered to be a new hallmark of cancer [13]. The changes of these metabolites represent important information about human diseases; multiple studies have used metabolomics-related techniques to find that the metabolic profiles of thyroid tumor tissue and normal tissue are different and found significantly upregulated or downregulated metabolites as an auxiliary diagnosis of thyroid potential biomarkers and therapeutic targets of cancer, and metabolic pathway analysis provides new ideas for exploring the mechanism of thyroid tumorigenesis and development [11, 14, 15]. There are also studies using gas chromatography-time-of-flight mass spectrometry (GC-TOF-MS) metabolomics technology to successfully screen six metabolites that can be used as oral squamous cell carcinoma saliva biomarkers in the oral squamous cell carcinoma [16]. In the field of endometrial cancer, a common gynecological malignancy, metabolomics was used to study related metabolites in patients through blood, urine, and other samples and found that related metabolites can be used as tumor markers and new therapeutic targets for early diagnosis and prediction recurrence and prognosis [17–19]. Therefore, metabolomics has unique advantages in studying the diagnosis and pathogenesis of human diseases, especially in the search for biomarkers, especially in the field of tumors [20–23].

Esophageal squamous cell carcinoma (ESCC) is the most common subtype of esophageal cancer in my country. The increase in the incidence of esophageal squamous cell carcinoma has led to a significant increase in the number of esophageal cancer cases in my country. In recent years, with the continuous maturity of metabolomics technology, many laboratories at home and abroad have used MRS or MS metabolomics technology to detect metabolic profiles in tissues, blood, or urine of patients with esophageal cancer; trying to the pathogenesis of esophageal cancer was found from the significantly changed metabolites [24–27]. Unfortunately, there is still no clear disease mechanism, but the results suggest that there are indeed changes in metabolites and metabolic pathways in the development of esophageal cancer, which accumulated important data for subsequent metabolomic research analysis, since the typical epidemiological survey of esophageal cancer shows that the incidence is related to the patient's diet, occupational habits, gender, age, and other factors [28–33]. And compared with MRS [34], MS can provide higher sensitivity for the identification and quantification of metabolites and reduce the complexity of metabolite separation; therefore, this study used HPLC-TOF-MS/MS technology. Differential metabolites were obtained by analyzing the tumor tissue and matched normal tissues of 210 patients with esophageal cancer in Zhenjiang, Jiangsu Province, a high-incidence area of esophageal cancer in China. Firstly, control tumor tissue and normal tissue as a single variable as much as possible to eliminate the influence of epidemiological factors to obtain common differential metabolites, and secondly, compare the experimental results obtained from other countries or regions as much as possible to obtain regional specific metabolites. Therefore, this experiment intends to analyze the differential metabolites of esophageal squamous cell carcinoma from the perspective of metabolomics, to find abnormal metabolic pathways, to explore potential biomarkers and therapeutic targets of esophageal cancer, and to provide a theoretical basis for the diagnosis and treatment of esophageal cancer.

## 2. Materials and Methods

### 2.1. Sample Collection

All patients were recruited from the Department of Cardiothoracic Surgery of the First People's Hospital of Zhenjiang City, Jiangsu Province, China. Tumor samples and clinical information were approved by the local Ethics Committee of Affiliated People's Hospital of Jiangsu University. All relevant ethical regulations involving human participants were followed. A total of 210 fresh primary tumor tissues and adjacent normal tissue samples were histopathologically confirmed as esophageal squamous cell carcinoma patients, which had not previously received any special antitumor treatment before surgery. The basic characteristics of the patients are shown in Table 2. The adjacent noncancerous tissues were more than 2 cm away from the tumor margin and pathologically confirmed as normal tissue at the resection margin. Tumor and normal tissues were loaded separately with tissue-protective fluid, frozen in liquid nitrogen, and then, stored in solid carbon dioxide at −80°C.

### 2.2. Sample Preparation

Samples previously stored at −80°C in solid carbon dioxide are transferred to a –80°C ultralow temperature refrigerator and then thawed for 30 minutes in a 4°C refrigerator. The cancer tissues from each sample are then accurately weighed and placed in liquid nitrogen for grinding and mixing. After mixing, it is then put in an EP tube to form a mixture of cancerous tissues. The adjacent tissues are treated in the same way as the cancer tissues.

### 2.3. Metabolite Extraction

All solid mixed samples were ground (6 replicates for cancer and adjacent tissues), and 50 mg was placed in a 2 mL centrifuge tube (50 mg solid sample was accurately weighed), and the metabolites were extracted using a 400 *μ*L methanol : water (4 : 1, *v*/*v*) solution with 0.02 mg/mL L-2-chlorophenyl alanine as an internal standard. The mixture was allowed to settle at -10°C before being treated with a high-throughput tissue crusher Wonbio-96c (Shanghai Wanbo Biotechnology Co., Ltd.) at 50 Hz for 6 min, then followed by ultrasound at 40 kHz for 30 min at 5°C. The samples were placed at -20°C for 30 min to precipitate proteins. After centrifugation at 13000 g at 4°C for 15 min, the supernatant was carefully transferred to sample vials for LC-MS/MS analysis. Then, 20 *μ*L of the supernatant was pipetted from all samples and mixed with the same volume to prepare a quality control sample (Quality control, QC). The volume of each QC was the same as that of the sample. It was processed and tested in the same way as the analysis sample, inserting a QC sample in every 4 samples to examine the repeatability of the entire analysis process.

### 2.4. UPLC-MS Analysis

Chromatographic separation of the metabolites was performed on an ExionLCTMAD system (AB Sciex, USA) equipped with an ACQUITY UPLC HSS T3 column (100 mm × 2.1 mm i.d., 1.8 *μ*m; waters, Milford, USA). The mobile phase consisted of 0.1% formic acid in water : acetonitrile (95 : 5, *v*/*v*) (solvent A) and 0.1% formic acid in acetonitrile : isopropanol : water (47.5 : 47.5, *v*/*v*)(solvent B). The solvent gradient changed according to the following conditions: from 0 to 0.5 min, 0% B to 0% B; from 0.5 to 2.5 min, 0% B to 25% B; from 2.5 to 9 min, 25% B to 100% B; from 9 to 13 min, 100% B to 100% B; from 13 to 13.1 min, 100% B to 0% B, and from 13.1 to 16 min, 0% B to 5% 0 for equilibrating the systems. The sample injection volume was 10 *μ*L, and the flow rate was set to 0.4 mL/min. The column temperature was maintained at 40°C. During the period of analysis, all these samples were stored at 4°C.

The UPLC system was coupled to a quadrupole time-of-flight mass spectrometer (Triple TOFTM5600+, AB Sciex, USA) equipped with an electrospray ionization (ESI) source operating in positive mode and negative mode. The optimal conditions were set as follows: source temperature, 550°C; curtain gas (CUR), 30 psi; ion source, GS1, and GS2, 50 psi; ion-spray voltage floating (ISVF), -4000 V in negative mode and 5000 V in positive mode, respectively; declustering potential, 80 V; collision energy (CE), 40 ± 20 V rolling for MS/MS; and cycle time, 510 mins. Data acquisition was performed in the data-dependent acquisition (DDA) mode. The detection was carried out over a mass range of 50-10002009*m*/*z*.

### 2.5. Metabolomic Data Analysis

#### 2.5.1. Data Preprocessing and Annotation

The components flowing out of the sample through chromatographic separation continuously enter the mass spectrum for continuous scanning for data acquisition, scanned once to obtain a mass spectrum, and then, added all ion intensities in all mass spectra to obtain the total ion current intensity.

After UPLC-TOF/MS analyses, the raw data were imported into the Progenesis QI 2.3 (Nonlinear Dynamics, Waters, USA) for peak detection and alignment. The preprocessing results generated a data matrix that consisted of the retention time (RT), mass-to-charge ratio (*m*/*z*) values, and peak intensity.

The mass spectra of these metabolic features were identified by searching reliable biochemical databases such as the human metabolome database (HMDB) (http://www.hmdb.ca/) and Metlin database (https://metlin.scripps.edu/) for accurate mass, MS/MS fragment spectra, and isotope ratio difference.

#### 2.5.2. Multivariate Statistical Analysis

The standard deviation of total expression in cancer tissues and tissues was compared and analyzed to determine if there was a significant difference at the metabolomics level.

A multivariate statistical analysis was performed using the ropls (Version1.6.2, http://bioconductor.org/packages/release/bioc/html/ropls.html) R package from Bioconductor on Majorbio Cloud Platform (https://cloud.majorbio.com), mainly including principal component analysis (PCA), partial least squares-discriminant analysis (PLS-DA), and orthogonal partial least squares discriminant analysis (OPLS-DA). PCA analysis was used for data overview and outlier detection. PLS-DA model was used to analyze the metabolite profile of cancer tissue and tissue samples. OPLS-DA removes the variation factors unrelated or orthogonal to the *Y* variable in *X* data variables; to better distinguish the differences, PLS-DA and OPLS-DA models were verified by 200 permutation tests to reveal the simple structure hidden behind the complex data.

#### 2.5.3. Differential Metabolites Analysis

After the optimal model was successfully constructed and selected, SciPy. Stats (Python package) (https://docs.scipy.org/doc/scipy/) was exploited to perform a one-way analysis of variance and fold change (FC) analysis on the detection results of cancer tissue and tissue samples using the OPLS-DA model. Combined with VIP value of more than 1 and *P* value less than 0.05 were further screened for target differential compounds, which were obtained by pattern recognition. Differential metabolite information was identified, and relevant metabolomics databases were consulted to screen and determine differential metabolites between the two groups.

The differential metabolites were clustered, and thermal images of metabolic profiles were used to show the overall trend of each metabolite between cancer tissues and tissues. The screened metabolites were used to establish a metabolic set with all metabolites of this species combined as the enrichment background for KEGG pathway enrichment analysis and topological analysis. The corrected *P* value was set lower than 0.05 to select the statistically significant result pathways.

#### 2.5.4. Validation of Potential Biomarkers

The receiver-operator characteristic curve (ROC curve) was used to analyze the similarity between cancer tissues and corresponding normal tissues for differential metabolites, and the discrimination ability of potential biomarkers was evaluated. The area under the ROC curve (AUC) > 0.9 and the 95% confidence interval (CI) of AUC calculated based on the nonparametric resampling method >0.8 were used as the screening criteria for diagnostic value.

### 2.6. Cer Transcriptomic Analysis Validation

#### 2.6.1. RNA Extraction, Library Preparation, and Illumina Hiseq Xten Sequencing

Total RNA was extracted from the tissue using Trizol Reagent according to the manufacturer's instructions (Invitrogen), and genomic DNA was removed using rDNase I RNase-free (Takara). RNA quality was verified using a 2100 Bioanalyzer (Agilent Technologies, Santa Clara, CA, USA) and the ND-2000 (NanoDrop Technologies). Only high-quality RNA samples (OD260/280 = 1.8 ~ 2.2, OD260/230 ≥ 2.0, RIN ≥ 8, 28S : 18S ≥ 1.0, >10 *μ*g) were used to construct the sequencing library. RNA-seq transcriptome strand library was prepared following the TruSeqTM Stranded Total RNA Library Prep Kit from Illumina (San Diego, CA) using 5 *μ*g of total RNA. Libraries were size selected for cDNA target fragments of 200–300 bp on 2% low-range ultra-agarose followed by PCR amplified using Phusion DNA polymerase (NEB) for 15 PCR cycles. After quantified by TBS380, the paired-end RNA-seq sequencing library was sequenced with the Illumina HiSeq Xten.

#### 2.6.2. Data Preprocessing and Quantification of Gene Expression Levels

The raw paired-end reads were trimmed and quality controlled by SeqPrep (https://github.com/jstjohn/SeqPrep) and Sickle (https://github.com/najoshi/sickle) with default parameters. Then, clean reads were separately aligned to the reference genome with orientation mode (GRCh38.p13, http://asia.ensembl.org/Homo_sapiens/Info/Index) using HIASAT (https://ccb.jhu.edu/software/hisat2/index.shtml) software. The mapped reads of each sample were assembled by StringTie (https://ccb.jhu.edu/software/stringtie/index.shtml?t=example) in a reference-based approach. And the expression level of each transcript was calculated according to the Transcripts Per Million reads (TPM) method based on the length of the gene and the number of reads mapped to the gene. RSEM (http://deweylab.biostat.wisc.edu/rsem/) was used to quantify gene abundances.

#### 2.6.3. Differential Expression Analysis and Functional Enrichment

Differential expression analysis was performed using the DESeq2. The *P* values were adjusted using Benjamini and Hochberg's approach for controlling the false discovery rate. Genes with fold change (FC) > 2 or <-2 and *P* value < 0.05 were considered to be significantly different expressed mRNAs (DEmRNAs). In addition, functional-enrichment analyses including GO and KEGG were performed to identify which DEGs were significantly enriched in GO terms and metabolic pathways at Bonferroni-corrected *P* value ≤ 0.05 compared with the whole-transcriptome background. GO functional enrichment and KEGG pathway analysis were carried out by Goatools (https://github.com/tanghaibao/Goatools) and KOBAS (http://kobas.cbi.pku.edu.cn/home.do).

#### 2.6.4. Combined Transcriptomics and Metabolomics Analysis

Fisher's exact test was used to carry out an enrichment analysis of the two omics to improve the reliability of the study and to identify the biological process most related to biological phenomena. At the same time, the KEGG pathway was visualized and analyzed, and the differential genes obtained by transcriptomics were compared with those obtained by transcriptomics. The differential metabolites obtained by metabolomics are also mapped to a KEGG pathway picture, so the differential metabolites and differential genes are enriched to explore whether Cer has a relationship with differential genes, and to explore the mechanism of its low expression. From the perspective of side verification, the authenticity of metabolomics data and the reliability of the above differential metabolites are candidate biomarkers for esophageal squamous cell carcinoma.

## 3. Results

### 3.1. Total Ion Chromatogram and Quality Control

**Figure 1:**
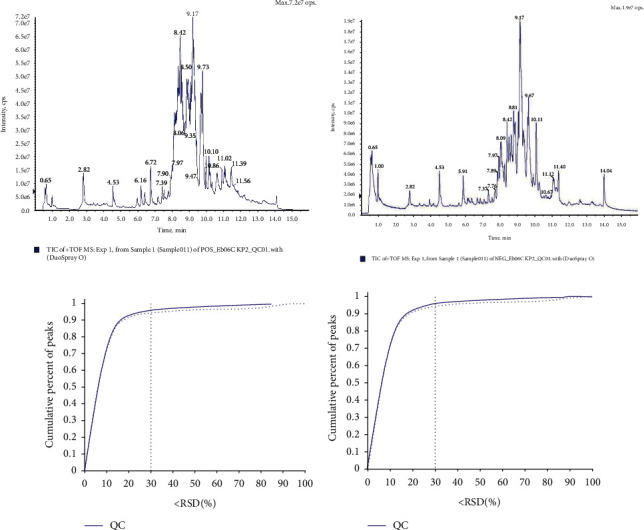
Quality control chart of control samples. (a) ESI (+) total ion chromatogram of control samples; (b) ESI (-) total ion chromatogram of control samples; abscissa is time; ordinate is the sum of ionic strength; (c) ESI (+) QC sample evaluation chart; (d) ESI (-) QC sample evaluation chart; note: RSD (%): standard deviation/mean value.

Here, we applied UPLC to obtain the metabolic profiles of the tissue of 210 normal and cancer in the positive and negative ESI models (Figure 1). In positive and negative ion mode, the total ion chromatogram of the quality control sample and the sample evaluation chart shows that under the detection conditions, the peak shape is good, and the distribution is relatively uniform (Figures 1(a) and 1(b)), and RSD < 0.3, and the proportion of peaks > 90% (Figures 1(c) and 1(b)). All data are indicated that the stability of the whole detection process and the data are excellent, and the obtained data could be used for the next analysis.

### 3.2. Sample Comparison and Pattern Discriminant Analysis

**Figure 2:**
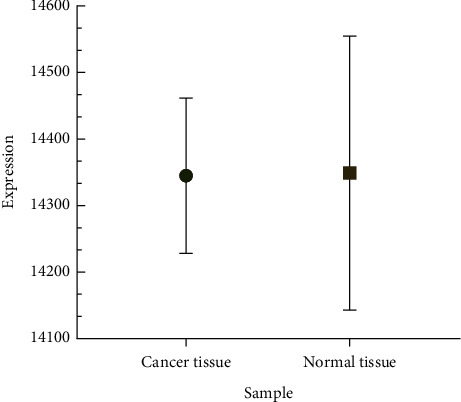
Comparison of total expression levels of metabolites between cancer and adjacent noncancerous tissues.

After preprocessing the raw data obtained by UPLC-MS, a total of 9,842 mass peaks were obtained (6482 in positive ion mode, 3360 in negative ion mode), and total expression and standard deviation and variance analysis of the expression data of 9842 ions were detected in all biological samples (cancer and adjacent tissues both had 6 biological replicates). The results (Figure 2) showed that the difference in the total expression of metabolites between cancer tissue samples and adjacent noncancerous tissue samples is small, and the *P* value is more than 0.05, indicating that the difference is not significant. It also indicated that the metabolites may be systematic in the two samples, and different metabolites have different expression trends in the tissues, resulting in metabolic balance. Therefore, it is necessary to further analyze the metabolites with different specific expressions between the experimental group and the control group.

**Figure 3:**
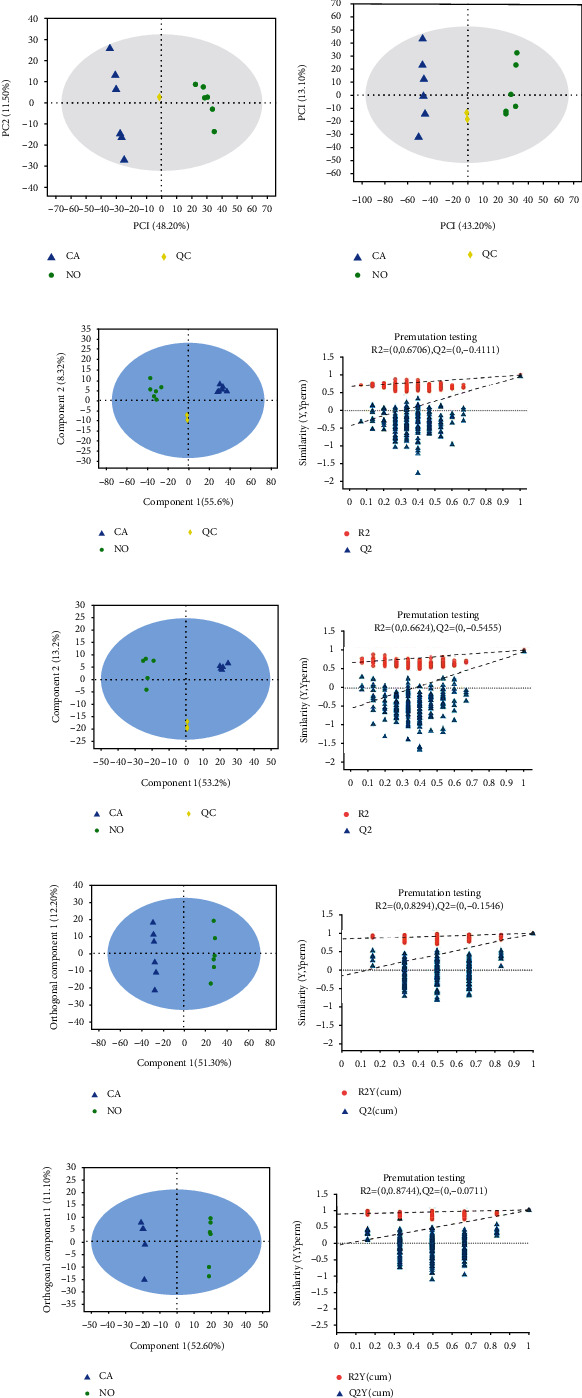
Multivariate analysis and identification by PCA, PLS-DA, and OPLS-DA. (a, b) Scatter plots analyzed by PCA; (c, e, g, i) PLS-DA (c, e) and OPLS-DA (g, i) showed that the metabolism of cancer tissues was different from that of adjacent tissues; (d, f, h, j) permutation test to test the reliability of the model. Note: CA: esophageal squamous cell carcinoma tissue; NO: control group (adjacent noncancerous tissue); QC: quality control sample.

Through the primary and secondary mass spectrometry data, the number of annotated cationic substances was finally 501 and the number of anionic substances was 334 in the library (self-built library, Metlin, HMDB, etc.). The PCA analysis was performed for this (Figures 3(a) and 3(b)). The results of QC samples were relatively concentrated, demonstrating that the whole process of analysis was stable, and there were significant differences between cancer and normal samples, with significant differences in metabolic profiles and good overall condition. The results of PLS-DA metabolite profiling (Figures 3(c) and 3(e)) show that there were significant differences in metabolomics data between cancer and adjacent noncancerous tissue in the cationic mode [R2X (cum) = 0.639, R2Y (cum) = 0.975, Q2 (cum) = 0.95] and the anionic mode [R2X (cum) = 0.664, R2Y (cum) = 0.949, Q2 (cum) = 0.932]. Both modes underwent 200 permutation tests, the results in cationic mode are shown in Figure 3(d), R2Y = 0.6702, Q2Y = −0.4111, the results in anionic mode are shown in Figure 3(f), R2Y = 0.6624, Q2Y = −0.5455; and Q2 regression line showed an upward trend in two modes, indicating that the model was robust and reliable, no overfitting occurred, and the PLS-DA model was effective. The results of further OPLS-DA analysis showed that there were no outlying samples and no significant within-group differences (Figures 3(g) and 3(i)). In the cationic mode (Figure 3(g)), the separation was better, and there were significant metabolomics differences [R2X (cum) = 0.635, R2Y (cum) = 0.998, Q2 (cum) = 0.984], 200 permutation tests were performed, and the results showed that R2 intercept was 0.8294, and Q2 intercept was -0.1546 (Figure 3(h)). Significant metabolic profile differences are also seen in the same anionic mode [R2X(cum) = 0.637, R2Y(cum) = 0.999, Q2(cum) = 0.986] (Figure 3(i)), the results of 200 permutation tests were shown in Figure 3(j), the R2 intercept was 0.8744, and the intercept of Q2 was -0.0711. The above-obtained results indicate that there was no “overfitting” of the model in the negative and positive ion models and that the model was reliable and could be used to distinguish tumors from normal tissue.

The comprehensive score plot showed that in more complex multivariate models, the PLS-DA model outperformed PCA in classification and separation, while the OPLS-DA model had higher discrimination and prediction rates than the former two.

### 3.3. Metabolic Pathway Analysis and Classification Annotation

**Figure 4:**
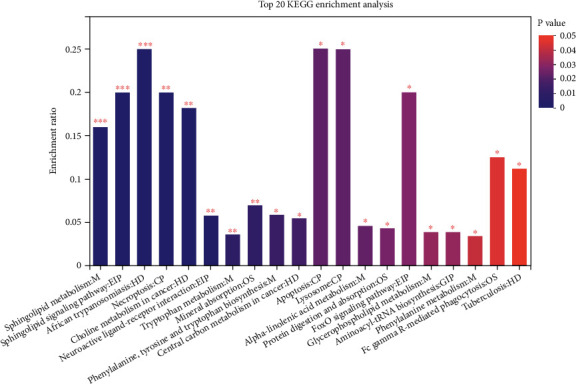
KEGG enrichment pathways of 685 metabolites with high activity. Note: the ordinate is the number of metabolites enriched, and the abscissa is the name of each metabolic pathway.

A total of 685 metabolites were annotated in the preprocessed dataset alignment HMDB 4.0 database, and all of them belonged to organic compounds in the first-level kingdom classification, and then, the annotated metabolites were classified according to different hierarchical levels (superclass, class, and subclass); names, the number of metabolites, and percentage of the selected HMDB hierarchy are displayed (Supplementary Table 1). And the classification of the same substance at different levels is different. After metabolic pathway analysis, ten pathways containing the highest number of identified metabolites were selected, indicating that metabolites are more active in these biological pathways (Figure 4); there are mainly neuroactive ligand-receptor interaction, sphingolipid metabolism, central carbon metabolism in cancer, linoleic acid metabolism, ABC transporter, protein digestion, and absorption. It is suggested that the above pathways play a key role in esophageal squamous cell carcinoma.

### 3.4. Differential Metabolite Identification Results

**Figure 5:**
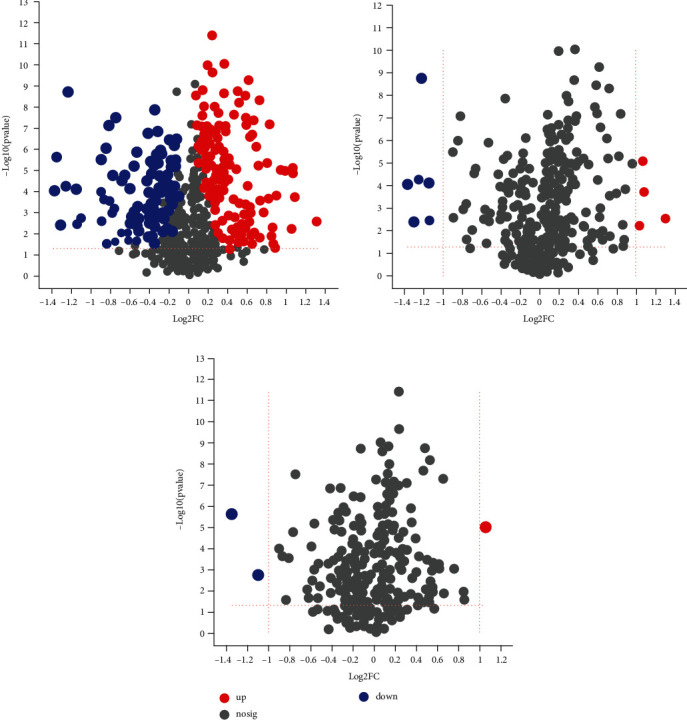
Volcano diagram of the difference between esophageal squamous cell carcinoma tissue and adjacent noncancerous tissue. (a) volcano plot of differential metabolites between esophageal squamous cell carcinoma tissue and adjacent noncancerous tissue; (b) volcano plot of representative differential metabolites in cationic mode; (c) volcano plot of representative differential metabolites in anion mode. Note: log2FC is the fold change value of the difference in the expression of metabolites between the two groups; -log10 (*P* value) is the statistics of the difference in the expression of metabolites.

**Figure 6:**
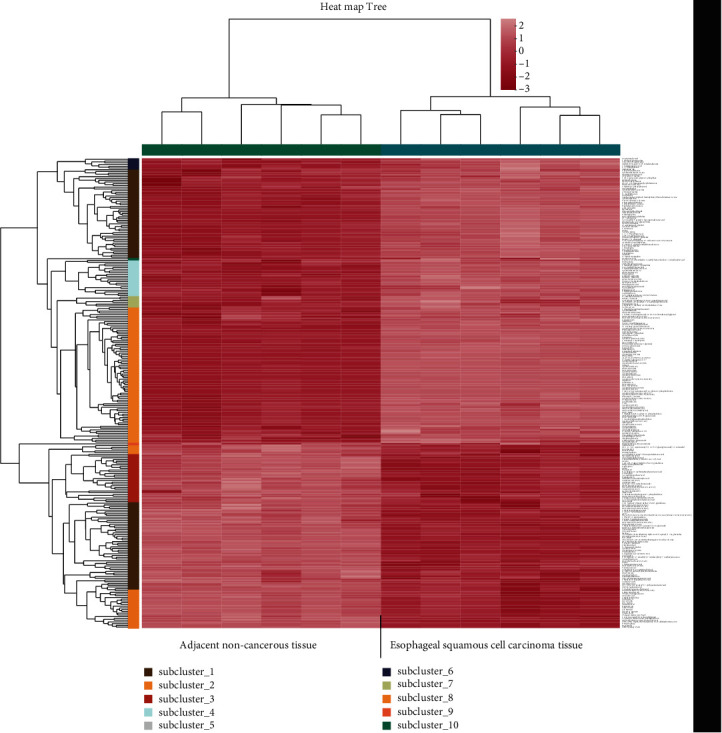
Cluster analysis of 269 differential metabolites between esophageal squamous cell carcinoma tissue and adjacent noncancerous tissue. Note: each column in the figure represents a sample, each row represents a metabolite, the right side is the name of the metabolite, and the upper side is the name of the sample. The color in the figure represents the relative expression level of the metabolites in this group of samples. For the changing trend of the specific expression level, see the numerical annotation under the color bar at the lower right.

**Figure 7:**
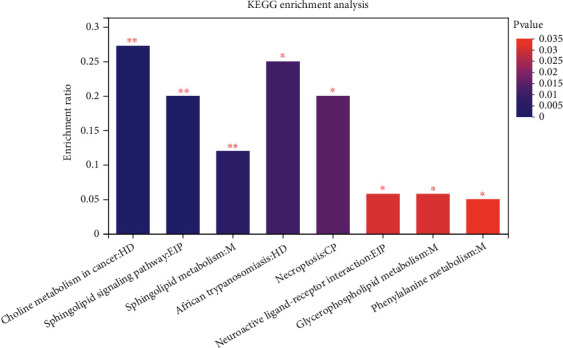
KEGG pathway enrichment analysis of 269 differential metabolites between esophageal squamous cell carcinoma tissue and adjacent noncancerous tissue. Note: the abscissa is the pathway name and the ordinate is the enrichment ratio, indicating the number of metabolites enriched in this pathway (metabolite number). The ratio of the number of metabolites annotated to the pathway (background number), the ratio, indicates the degree of enrichment. The column color gradient indicates the significance of enrichment, and the darker the default color, the more significant the enrichment represents this KEGG term, where those with *P* value or FDR < 0.01 are labeled as ∗ and those with *P* value or FDR < 0.05 are labeled as ∗.

**Figure 8:**
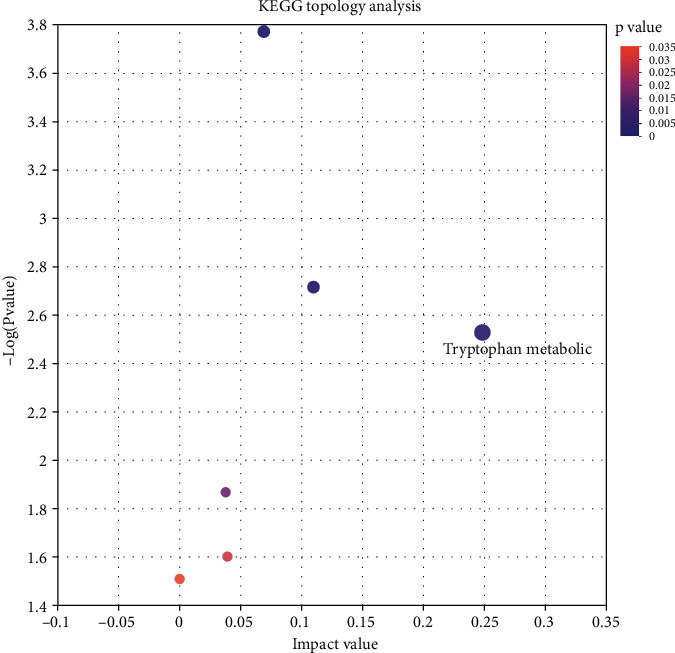
Bubble diagram of KEGG pathway topological analysis. Note: each bubble indicates a KEGG pathway; the horizontal axis indicates the relative importance of metabolites in the pathway impact value; the vertical axis indicates the enrichment significance-log10 (*P* value) of metabolites involved in the pathway; the bubble size represents the impact value; the bubble indicates the importance of the pathway.

After the screening of the above conditions, a total of 269 differential metabolites were obtained under the negative and positive ion mode in the OPLS-DA model, and 164 metabolites were upregulated, and 105 were downregulated (Figure 5(a)). The cluster analysis of metabolites was continued for these differential metabolites, showing that the trend of metabolite expression was different between the two groups of samples; the expression pattern was quite different obviously (Figure 6); more importantly, KEGG pathway analysis enriched 48 channels, and 8 metabolic pathways were obtained after setting the *P* value less than 0.05, and the analysis results showed that the significant metabolic differences of ESCC tissues and normal tissues were involved in choline metabolism (in cancer), sphingolipid metabolism, glycerophospholipid metabolism, sphingolipid signaling pathway, phenylalanine metabolism, neuroactive ligand-receptor interaction, and the formation of necroptosis and African trypanosomiasis (Figure 7), which may be related to tumor pathogenesis, and the topological analysis results showed that tryptophan metabolism, glycerophospholipid metabolism, sphingolipid metabolism, and other pathways were significant abnormalities in ESCC, with topological analysis of the tryptophan metabolic pathway having the strongest influence (Table 3, Figure 8).

### 3.5. Potential Biomarker

Increasing the screening criteria FC to greater than 2 resulted in representative differential metabolites (Figures 5(b) and 5(c)), with 10 representative differential metabolisms in the cationic mode, 3 in the anionic mode, and in both modes, the number of metabolites upregulated was 8 and downregulated was 5, and these metabolites could be analyzed as potential diagnostic candidates (Table 4), including 8 lipids and their related metabolites, 2 alkaloids and derivatives, 2 organic acids and derivatives, and 1 organic heterocyclic compound.

**Figure 9:**
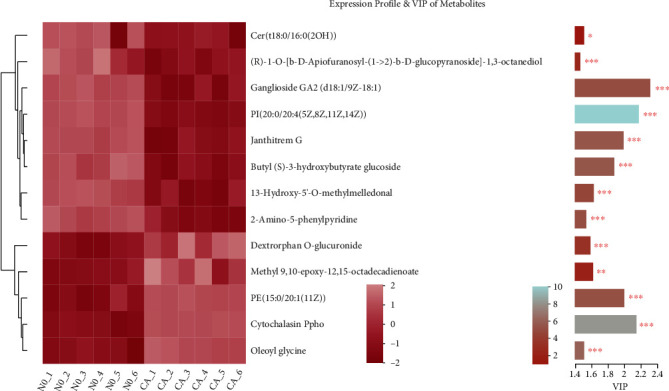
Expression profile and VIP of metabolites. CA: esophageal squamous cell carcinoma; NO: adjacent noncancerous tissue. Note: the cluster dendrogram of metabolites is shown on the left side. Each row indicates a metabolite. The color indicates the relative expression of this metabolite in this group of samples. The bar color indicates significant differences in metabolites in the two groups, namely, *P* value, the smaller the *P* value, the darker the color.

Clustering and VIP analysis of the 13 metabolites with significant differences (Figure 9) showed that the expression patterns of the 13 metabolites were quite different between cancer tissues and normal tissues, with ganglioside GA2 (d18 : 1/9Z-18 : 1) having the highest VIP value, indicating that it contributed the most important to the differences between the two groups.

**Figure 10:**
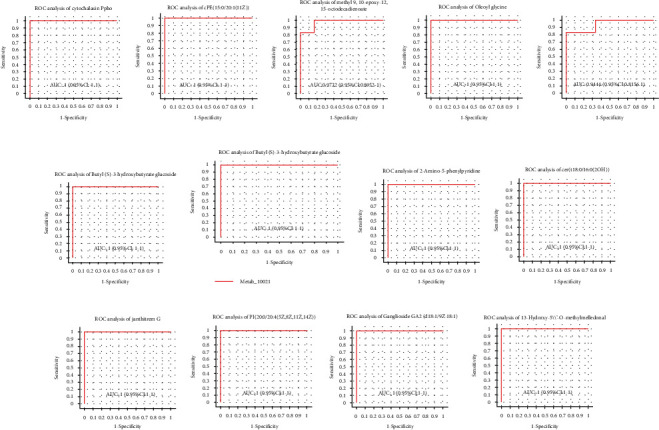
Plot of ROC curve analysis for 13 significant differential metabolites. (a–m) [(a) cytochalasin Ppho; (b) PE (15:0/20:1(11Z)); (c) methyl 9,10-epoxy-12,15-octadecadienoate; (d) oleoyl glycine; (e) dextrorphan O-glucuronide; (f) butyl (S)-3-hydroxybutyrate glucoside; (g) (R)-1-O-[b-D-apiofuranosyl-(1->2)-b-D-glucopyranoside]-1,3-octanediol; (h) 2-amino-5-phenylpyridine; (i) Cer (t18:0/16:0(2OH)); (j) janthitrem G; (k) PI (20:0/20:4(5Z,8Z,11Z,14Z)); (l) ganglioside GA2 (d18:1/9Z-18:1); (m) 13-hydroxy-5′-O-methylmelledonal]. Note: The *X*-axis is 1-specificity; *Y*-axis is sensitivity; the point indicated on the curve is the optimal critical value; the “bar” on the point is the corresponding point confidence interval of specificity and sensitivity; AUC indicated in the figure is the area under the corresponding curve.

The receiver-operator characteristic curve (ROC curve) was used to deeply evaluate and analyze the differential ability of the above 13 potential biomarkers.  Figure 10 showed that the area under the curve (AUC) of all metabolites was greater than 0.9, and the 95% confidence interval (CI) was greater than 0.8. Therefore, these 13 metabolites with different expressions had the potential to distinguish ESCC samples from the normal samples and could be used as potential metabolic biomarkers;

### 3.6. Transcriptome Data Overview

**Figure 11:**
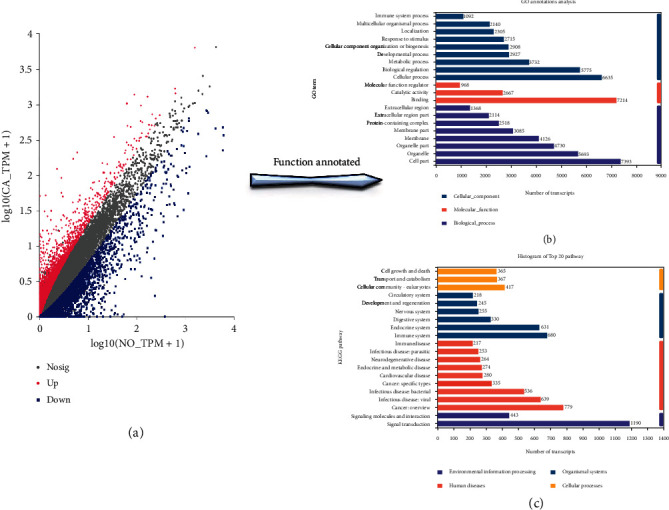
Differential gene expression scatter plot and functional annotation top 20 pathway histogram. (a) Differential gene expression scatter plot; (b) top 20 pathways by GO annotation; (c) top 20 pathways by KEGG annotation.

**Figure 12:**
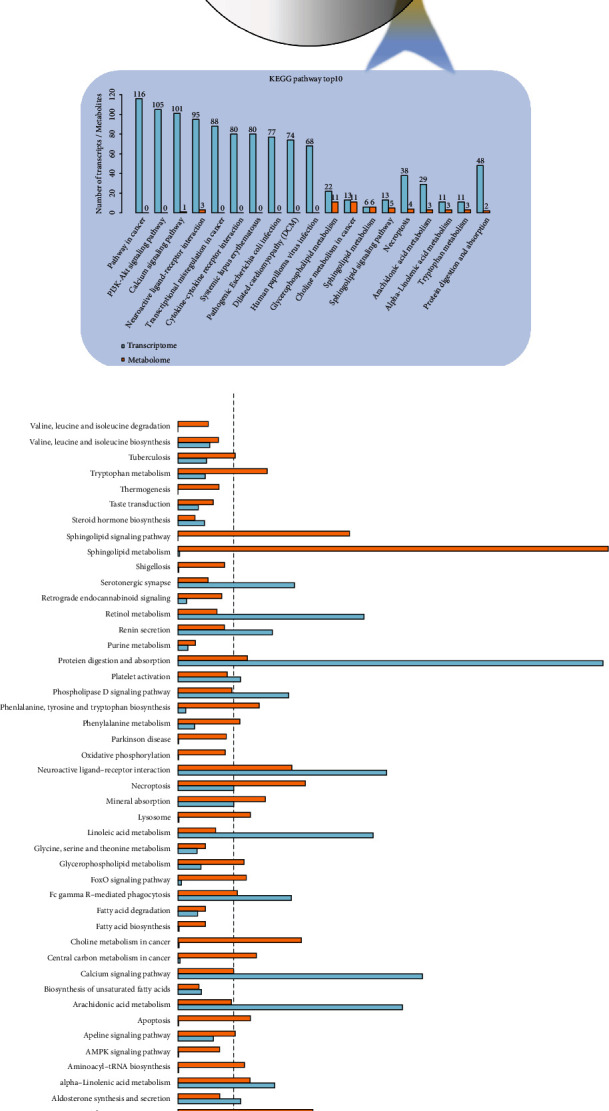
KEGG pathway comparative analysis and enrichment analysis of differential gene/metabolite. (a) Comparative analysis of KEGG pathway; (b) KEGG enrichment analysis histogram. Note: each column in the figure represents a KEGG pathway, different colors represent different omics, blue represents the transcriptome, and yellow represents the metabolome; the higher the column, the more active the biological pathway is in the tested samples.

The RNA quality of the sample was qualified, and the sequencing quality was high. After data preprocessing, the mapping efficiency was high. A total of 84,630 transcripts were obtained for differential analysis. Using DESeq2 software, with |log2FC| > 1, *P* adjust < 0.05 as differential gene screening criteria for differential analysis, 8549 differential mRNAs (DEmRNA) were obtained, and 4138 mRNAs were upregulated, and 4366 mRNAs were downregulated on tumors. The screened major DE mRNAs were annotated by GO and KEGG, and a total of 55 GO pathways and 187 KEGG metabolic pathways were annotated (Figure 11). The biological process (BP) pathways mainly annotated by GO are cellular process (GO:0009987) and biological regulation (GO:0065007), indicating that these differential genes are mainly used to regulate biological processes; the functional pathway with the most DE mRNAs annotated by KEGG is the protein digestion and absorption pathway (map04974) in the secondary category digestive system, including 113 transcripts, indicating that the occurrence of esophageal squamous cell carcinoma has the greatest impact on the functions related to protein digestion and absorption. It may be closely related to the clinical features of the patient's significant weight loss in the later stage of the tumor (Figure 12).

### 3.7. Transcription-Metabolism Combined Omics Analysis of Cer Metabolic Pathway

Using R language, the obtained differential genes and differential metabolites are used for KEGG pathway comparative analysis and enrichment analysis, which not only compares the pathways involved in genes in the transcriptome and the pathways involved in metabolites in the metabolome but obtains the number of commonly involved pathways and also identifies the top 10 KEGG pathways with the largest number of identified differential genes, and differential metabolites were identified to identify the biological processes most related to biological phenomena (Figure 12). As shown in Figure 12(b), it can be seen that there are more metabolites including Cer enriched in the sphingosine pathway and the most enriched genes in the protein digestion and absorption pathway.

**Figure 13:**
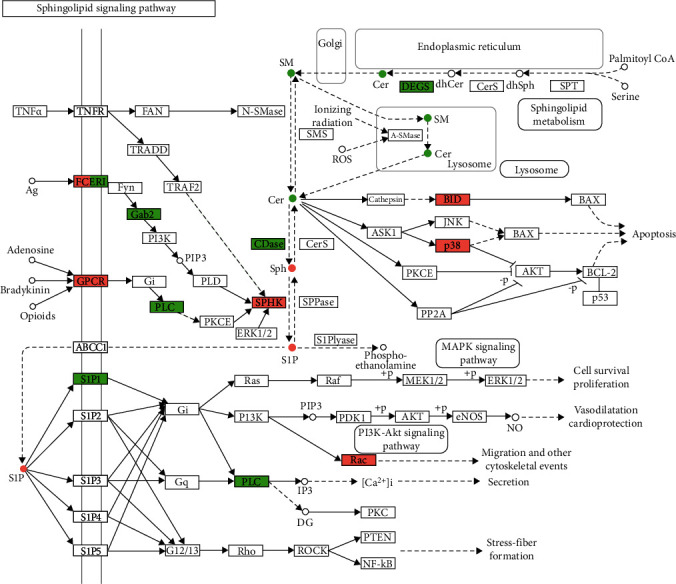
Sphingolipid metabolism pathway visualization. Note: green indicates downregulation; red indicates upregulation.

Cer is one of the important lipids in esophageal squamous cell carcinoma. The enrichment analysis of 13 representative differential metabolites found that only Cer involved in the sphingolipid metabolism pathway was significant (Figure 12(b)). Therefore, we took Cer as the representative substance of this joint transcription-metabolism analysis. We analyzed the sphingolipid metabolism pathway in which CER participates through a joint transcription-metabolism analysis and mapped the differential genes obtained by transcriptomics and the differential metabolites obtained by metabolomics into a pathway picture at the same time, which can integrate pathway data intuitively and comprehensively (Figure 13). Firstly, it is dehydrogenated from dihydroceramide (dhCer) by DEGS and then forms SM through Golgi. SM enters lysosomes and can be converted into lysosomes by acid sphingomyelinase (ASMase). Cer is transported out of lysosomes and converted to sphingosine by cytosine deaminase (CDase), while sphingosine can also be converted back to Cer by ceramide synthase (Cers); secondly, Cer plays an indispensable role in the formation of cancer. For example, Cer affects apoptosis through the synthesis of p38 and can also be converted into sphingosine to further affect the MAPK signal pathway and PI3K-Akt signal pathway. Previous studies have shown that these signal pathways are critical for tumors. Metabolomics data shows that Cer (FC: 0.419) and SM (FC: 0.57) are downregulated in tumors; sphingosine (FC: 1.08) and sphingosine 1-phosphate (FC: 1.41) are upregulated. From the transcriptomic data, the expressions of DEGS2 (FC:0.27) and CDase (FC:0.09) were downregulated, while the expressions of SMS, CerS, and A-SMases, which are related to Cer synthesis and metabolism, did not change significantly. Therefore, the decreased expression of Cer in esophageal squamous cell carcinoma is due to less synthesis (decreased expression of DEGS2) and increased metabolism (increased expression of Sph and SIP). And we also enriched the sphingolipid biosynthetic process (GO: 0030148) in the GO enrichment analysis of DEmRNAs. Therefore, we believe that the transcriptome analysis results can indirectly confirm the authenticity and reliability of the metabolomic data from an upstream perspective.

## 4. Discussion

Esophageal squamous cell carcinoma (ESCC) is a complex malignant tumor with a low degree of differentiation, high degree of malignancy, rapid growth rate, and a mortality rate of up to 90%. By researching the metabolic state of malignant ESCC tissues, we can obtain local information on pathologically altered tissues and gain insight into the biological processes of ESCC. Therefore, to obtain the common and universal differential metabolites of ESCC in this study, large sample data were used to analyze the metabolic profiles of paired tumor tissues using HPLC-TOF-MS/MS technology to eliminate differences in age and gender. At the same time, because esophageal squamous cell carcinoma has obvious localized high incidence and epidemiological characteristics related to regional dietary structure [7], only patients in Zhenjiang, Jiangsu Province, was selected, and the region was taken as a large variable, to obtain region-specific data representative metabolites. From the analysis results, it was found that the metabolic profiles of ESCC tissues and paired normal tissues were significantly different. 685 metabolites were annotated and active in the metabolic pathway of glycerophospholipids after KEGG pathway analysis. Then, OPLS-DA pattern discriminant analysis showed that 269 metabolites were differentially expressed, and they were enriched into 48 metabolic pathways. After screening by P, 8 metabolic pathways were significant, most of them were amino acid metabolism or lipid metabolism. The topological analysis found that the tryptophan metabolic pathway had the highest impact value; to further discover candidate metabolites with specificity, sensitivity, and potential diagnostic significance, the screening criteria were increased to FC > 2 and the discrimination ability was evaluated and analyzed. 13 biological metabolites were found to be potential metabolic biomarkers to differentiate ESCC tissues from normal tissues. And they have high confidence.

Cytochalasin was first isolated as a metabolite from the long peristaltic genus when Tel and colleague Turner screened anticancer drugs in the drug laboratory in 1964 [35]; more than ten species have been found so far, with the main effects of inhibiting cytoplasmic division, inhibiting cell motility and phagocytosis [36]. Both cytochalasin B (CB) and cytochalasin D (CD) can induce malignant cells including human esophageal cancer to form multinucleated cells, which are teratogenic [37–40]. The results of this study found that the cytochalasin Ppho level in the tumor cytochalasin family was increased, which has similar chemical structures as CB and CD. Somers and Murphey found that the coincidence rate between CB multinucleated effects and carcinogenesis in vivo was extremely large, indicating that cytochalasin is associated with tumor formation [41]; later study has found that four cytochalasins inhibit the energy metabolism of tumor cells through the creatine metabolism pathway, thereby playing an effective antiproliferative effect, such as cytochalasin Q (CQ) [42]; cytochalasin P-1 was found to be cytotoxic in four tumor cells from the marine fungus Charcoal sp., such as hepatic carcinoma and lung cancer [43]. Therefore, whether cytochalasin is an advantage or disadvantage for tumor formation, the specific mechanism is still unknown. In the results of this study, it was found that the level of cytochalasin Ppho (CP) in the cytochalasin family was increased in tumor tissues compared with normal tissues (Table 4). It has a chemical structure similar to CB, CD, and CQ. The relationship between CP and cancer has not been reported (including esophageal cancer). However, in this experiment, it was found that it was significantly highly expressed in ESCC, suggesting that it may have a similar function to CB, leading to the occurrence of ESCC, or is one of the immune substances in the human antitumor generation, like CQ. From the analysis of metabolomic data, it can be concluded that CP has the potential as a biomarker to distinguish cancer tissue from normal tissue, which can be used for the subsequent detection of esophageal squamous cell carcinoma. Dextrorphan O-glucuronide, which is the same alkaloid and its derivatives, is a natural human drug metabolite of dextrorphan produced by UDP-glucuronosyltransferases (UGTs) in the liver, and dextrorphan is commonly used for analgesia and belongs to the antipyretic and analgesic drugs [44], so it is not an endogenous metabolite and cannot be used as an endogenous metabolic biomarker to diagnose esophageal cancer. Interestingly, in the results of this experiment, the content of dextrorphan O-glucuronide in cancer tissues is significantly higher than that in normal tissues (Table 4), and it was speculated that the reason for its high expression may be that dextrorphan taken by cancer patients due to pain tends to act on cancer tissues when they are metabolized *in vivo*, suggesting that dextrorphan may have the potential to be used as a drug carrier for targeting esophageal cancer.

Both PE and PI belong to the class of glycerophospholipids, which are the main components of biofilms and are involved in regulating a variety of life activity processes. Increasingly, basic studies have found that abnormal lipid metabolism may be an important factor in the occurrence and development of malignant tumors and is the potential pathogenesis of a variety of tumors [45, 46]. In this study, we found that glycerophospholipid metabolism was abnormal in cancer tissues (Table 3), and the expression of PE in cancer tissues was higher than in normal tissues, while PI was opposite (Table 4), speculating that it may be related to the metabolic pathway because it was found that the interconversion between the metabolism of different phospholipids, including PI and PE, constitutes a complex phospholipid metabolic network (see the left side of Figure 12), for example, phosphatidic acid (PA), as an important phospholipid intermediate reactant or intermediate product, could generate PE and PI, while PE could generate phosphatidylcholine (PC) through PEMT pathway, and PC could interconvert with PS (phosphatidylserine) and sphingomyelin (SM), indicating that abnormal PC metabolism is a major marker of cancer cells. Studies in a variety of cancers, including ovarian and endometrial cancer, have shown that PCS may differ between carcinomas and adjacent tissues [47, 48]; for example, the expression of PC was significantly downregulated in squamous cervical cancer (SCC) patients compared with uterine fibroids (UF) patients [49]. So, it is believed that the PC metabolic pathway involved in PE may be necessary for tumor growth and development, while the main pathway of PC production is the conversion from PE to PC by the PEMT enzyme. PEMT gene expression and activity are downregulated in patients with hepatic carcinoma [50], and PEMT expression is significantly upregulated in patients with non-small-cell lung cancer [51]. It indicates that the expression of phospholipid metabolism in different types of tumors does not necessarily have a unique direction. Therefore, it can be further speculated that PEMT enzyme activity may be decreased in esophageal cancer, and abnormal PC metabolism leads to the occurrence of esophageal cancer. Some studies have suggested that PC metabolism-related metabolic enzymes have the potential to act through or associate with estrogen and its receptors [47, 48], and the specific mechanism has not been clarified. Mass spectrometry results show that the expression of PI in cancer tissues is reduced (Table 4), PI exists in three alternative forms *in vivo*, PI, phosphatidylinositol 4-phosphate (PIP), and phosphatidylinositol 4,5-diphosphate (PIP2), and the reason for the downregulation of expression may be that multiple factors lead to the formation of various products such as PIP/PIP2 during cancer tissue cell proliferation, which is involved in the occurrence of cancer, and the expression of PI3K enzyme content is significantly increased in esophageal squamous cell carcinoma [52–54]; PI3K can phosphorylate the third carbon atom on the PI inositol ring so that the formed products have the functions of promoting cell proliferation and enhancing resistance to apoptosis, which have important effects on the occurrence and development of cancer. Multiple studies have shown that increased PI metabolism is closely related to growth [55, 56]. The decrease in the expression of PI in this study reflects the increase in the content of the PI3K enzyme. Therefore, it confirms the results of other studies. It can also be proposed to use PI and PI3K enzymes as a combined marker to jointly diagnose ESCC to achieve higher sensitivity and accuracy and precision medical testing.

In addition to phospholipids, lipids also include sphingolipids, such as ceramide (Cer) and sphingomyelin (SM), and mass spectrometry results show that ceramide levels are decreased, and sphingolipid metabolism is abnormal in tumor tissues compared with normal tissues (Table 3 and Table 4). Cer is closely related to cell growth, senescence, apoptosis, and other life cycle-related activities and is considered to be one of the tumor inhibitors [57]. Abnormal expression of ceramide has been found in many cancer tissues. In ovarian cancer, it is found that the total Cer content of cancer tissues is reduced compared with paired normal tissues; thus, reducing the apoptosis of tumor cells and promoting tumor development, as well as Cer in clinical samples, the reduction is associated with malignancy and poor prognosis of astrocytomas [58]. In head and neck squamous cell carcinoma, the expression of cl8-Cer is significantly reduced [59]; in addition, Cer metabolism-related enzymes, such as neutral SM hydrolase 2 (nSMase2 or SMPD3), can increase the level of Cer by hydrolyzing SM to inhibit cell proliferation and prolong cell cycle. However, the relationship between esophageal squamous cell carcinoma and ceramide has not been supported by the literature. From the results of this study, it can be known that the content of Cer (t18:0/16 : 0(2OH)) in esophageal squamous cell carcinoma is lower than that in matched normal tissues. Therefore, combined with the expression of Cer in other cancer tissues, it is proposed that the total Cer content in tissues is one of the criteria for judging ESCC and adjacent cancer [60, 61]. At the same time, the results of transcriptome and metabolomic analysis showed that the inhibition of Cer anabolic enzymes and the activation of catabolic enzymes led to the loss of Cer in esophageal cancer tissues, suggesting that the loss of Cer anabolic enzyme expression in esophageal squamous cell carcinoma is passive selection during disease progression.

Gangliosides (GLS) are sialic acid-containing glycosphingolipids distributed on the surface of various tissue cells. It is located on the cell membrane mainly by inserting the hydrophobic ceramide part into the outer plasma membrane of the lipid double molecule layer, and its oligosaccharide part is exposed to the cell surface, which can interact with other components of the cell membrane, such as adhesion molecules and receptors [62], regulate receptor-mediated signal transmembrane transmission, and thus, regulate cell differentiation and motility and migration. GLS is involved in tumor cell metastasis and development [63, 64]. Monosialoganglioside GM2 in GLS can downregulate the phosphorylation of EGFR at position 1045, inhibit the phosphorylation of growth factors and the activation of growth factors, and then, inhibit the motility and migration of tumor cells [64–66]; GM2 (also known as GA2) expression level is lower in cancer tissues, and the ability to inhibit cell growth is poor. At the same time, it can block the information transmission of nucleic acid factors in the early stage of tumor necrosis factor (TNF) transcription, interrupt the information transmission, and inhibit the production of TNF by cells. The simultaneous action of the two leads to abnormal cell proliferation and weakens the immune capacity of patients [67]. There have been early reports on the use of the GM3 : GD3 ratio as an indicator for early diagnosis of melanoma and dynamic observation of efficacy [68], indicating that it is feasible to use ganglioside GM2 as a clinical diagnosis; therefore, in this study, it was found that the content of ganglioside GA2 (also called ganglioside GM2, GM2) was reduced (Table 4), and the ability to inhibit cell growth was weakened, leading to the occurrence of ESCC. Therefore, the content of GM2 in tissue can be used as one of the markers for the diagnosis of esophageal cancer, and GM2 may also become one of the targets of ESCC therapy.

The significant differential metabolites belonging to both lipids and analogs also include 13-hydroxy-5′-O-methylmelledona, which is a class of lipids that exist in both the cell membrane system, intracellular and extracellular, and widely distributed [69, 70]; 13-hydroxy-5′-O-methylmelledona is involved in many cell life activities and has a certain inhibitory effect on cell proliferation [69]. In this study, it was found that the expression level of 13-hydroxy-5′-O-methylmelledona in cancer tissues was lower than that in normal tissues (Table 4). It was speculated that 13-hydroxy-5′-O-methylmelledona may have peroxidation of free radicals in cancer tissues, resulting in high concentrations of lipid peroxide (LPO), inducing cellular DNA damage which causes cellular senescence and tumor production, resulting in its decreased expression; meanwhile, studies have demonstrated that the concentration of LPO in thyroid cancer tissues and esophageal cancer cells is indeed significantly higher than that in normal tissues [70–74]. Significant differential metabolites that are also involved in lipid peroxidation and have cell membrane stabilizing functions are butyl (S)-3-hydroxybutyrate glucoside and (R)-1-O-[b-D-apiofuranosyl-(1->2)-b-D-glucopyranoside]-1,3-octanedio (Table 4) [69], both of which are present in body fluids and on cell membranes and can be excreted through urine; the ROC analysis of both is greater than 0.9, and the results are good. It is speculated that the three lipids can be used as biomarkers and combined analysis of LPO levels in tissues to distinguish esophageal cancer from adjacent tissues.

Oleoyl glycine (OLGly) is upregulated in esophageal cancer tissues by HPLC-MS results (Table 4). It is an endogenous organic acid and an analog distributed on the cell membrane [70] and has biological effects such as regulating body temperature and exercise in rats [75] and increasing insulin sensitivity [76]. Metabolomics examination of secretions from patients with colorectal cancer revealed that OLGly was abnormally expressed in the feces of patients with cancer and was considered to be associated with cancer [77]. OLGly treatment of cell experiments revealed that OLGly could dose-dependently promote protein production to increase total cellular protein content [78], speculating that the increased expression of OLGly in esophageal cancer tissues endogenously promotes protein synthesis and provides raw materials for the continuous proliferation of cancer cells. Therefore, in future research, we can focus on whether the occurrence of esophageal cancer is related to the increase of OLGly. If so, OLGly can be used as both a marker for the diagnosis of esophageal cancer and a target for treatment.

The differential metabolites belonging to organic acids and analogs also include Janthitrem G, which is structurally similar to NLF-ii, is involved in the process of cell signal transduction, and has the effect of a cell membrane stabilizer. The results showed that the expression in cancer tissues was lower than that in normal tissues (Table 4). Some studies predicted that it was related to epilepsy caused by neoplastic effects on the brain, spinal cord, or peripheral nerves, resulting in sudden and involuntary skeletal muscle contraction of the brain or brainstem origin [70]. But its association with the mechanism of tumorigenesis has not been specifically studied.

In esophageal cancer tissues, 2-amino-5-phenylpyridine (Phe-P-1, 2-APP) was found to be downregulated, as it belongs to an endogenous mutagenic aromatic amine in the metabolism of phenylalanine in the differential metabolic pathway [79]; it is a pyrolysis product of phenylalanine in protein, which is mutagenic to strains and can be used as an initiator for the second-stage mouse skin model [80], and is similar in structure to 4-aminobiphenyl (4-ABP), an aromatic amine carcinogen that can induce human bladder cancer [81]. It is speculated that it may also interact with DNA leading to the occurrence of cancer resulting in reduced expression [82].

Methyl 9, 10-epoxy-12, 15-octadecadienoate have not yet been reported, but it was first found to be upregulated in esophageal cancer in this experiment.

## 5. Conclusion

**Figure 14:**
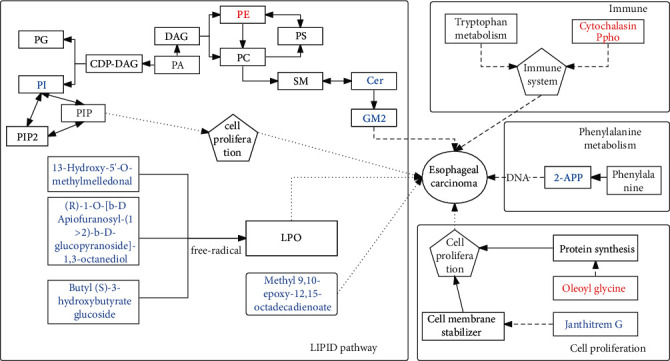
Metabolomics reprogramming in esophageal squamous cell carcinoma. , inhibition relationship; , promote relationships; metabolites in blue font indicate decreased expression levels of metabolites; metabolites in red font indicate increased expression levels of metabolites; DAG: diacylglycerol.

The metabolic complexity of ESCC is evident, affecting myriad metabolites and metabolic pathways. Therefore, we believe that relying on a metabolic biomarker to comprehensively reflect the pathological state of ESCC is difficult to provide a high enough diagnostic value. Therefore, combined biomarkers are the trend in the future diagnosis of ESCC [83, 84]. And its successful application in the prediction of colorectal cancer and urinary bladder cancer has been reported [84, 85]. Multiomics technology can perform large-scale and comprehensive detection of tissue, blood, and other samples, so it is inevitable to use multiomics technology to find potential biomarkers. From the differences in the metabolic profiles of esophageal squamous cell carcinoma tumor tissue and normal tissue in this experiment, we learned that, as a metabolic disease, esophageal squamous cell carcinoma is closely related to the metabolic reprogramming and metabolic pathways of various substances.  Figure 14 shows that 12 potential biomarkers that are significantly upregulated (red) or downregulated (blue) are mainly related to biological processes such as lipid metabolism, immune-related regulation, phenylalanine metabolic pathways, and cell proliferation [86, 87]. Importantly, we found that these twelve significantly different substances have the potential to be diagnostic biomarkers for ESCC in the future, and transcriptomic analysis also confirmed the reliability of the metabolic profile. However, there is still a considerable distance for differential metabolites to become biomarkers or combined biomarkers for clinical use, but our study provides a preliminary validated candidate for the next biomarker research. In addition, this study further explored the metabolic reprogramming characteristics of esophageal squamous cell carcinoma in high-incidence areas of China, which has reference significance for exploring the pathogenesis of esophageal squamous cell carcinoma.

## Figures and Tables

**Table 1:** Estimated number of new and death cases in high-incidence countries in 2020, both sexes and all ages.

Estimated number of new and death cases in high-incidence countries				
Country	New cases	Morbidity	Death cases	Mortality
China	324422	53.75%	301135	55.30%
India	63180	10.50%	58342	10.70%
Japan	26262	4.30%	12270	2.30%
Bangladesh	21745	3.60%	20319	3.70%
United States of America	18309	3%	16209	3%
Others	150182	24.90%	135801	25%

Note: data cited from GLOBOCAN2020 database (IARC website, http://gco.iarc.fr/today).

**Table 2:** Clinical sample characteristic information statistics.

Characteristic	Quantity	Percentage %
Gender		
Male	159	75.7
Female	51	24.3
Age		
*n* < 60	36	17.14
60 ≤ *n* < 70	102	48.57
*n* ≥ 70	72	34.29
Smoking status		
Ever	90	42.9
Never	120	57.1
BMI		
Underweight	18	8.57
Normal	157	74.76
Overweight	26	12.38
Obese	9	4.29

**Table 3:** KEGG pathway topology analysis detailed table.

Pathway description	Match-status (*A*|*B*)	Impact value	*P* value corrected
Tryptophan metabolism	3|56	0.2481	0.01486
Glycerophospholipid metabolism	3|48	0.1101	0.01284
Sphingolipid metabolism	3|21	0.0691	0.001685
Phenylalanine metabolizes	2|46	0.03904	0.08317
Phenylalanine, tyrosine, and tryptophan biosynthesis	2|33	0.03785	0.05449
Purine metabolism	2|81	0.02886	0.1624
Steroid hormone synthesis	2|89	0.01454	0.1506

Note: match-status indicates the situation that which the metabolites are involved in the pathway. *A* indicates the number of metabolites involved in the pathway in the current metabolic set, and *B* indicates the total number of metabolites in the current pathway; impact value: the comprehensive importance score of the pathway, with a total score of 1; *P* value corrected value is the corrected of the enrichment significance *P* value of metabolites involved in the pathway.

**Table 4:** Representative differential metabolites between esophageal squamous cell carcinoma tissue and adjacent noncancerous tissue.

Number	Metabolite	Formula	ID	Retention time	*M*/*Z*	VIP	*P*	Exp	FC
1	Cytochalasin Ppho	C30H41NO6	HMDB0035368	7.30	510.28	2.31	0.00001057	↑	2.087
2	PE (15:0/20:1(11Z))	C40H78NO8P	HMDB0008900	11.91	732.55	2.21	0.0001900	↑	2.125
3	Methyl 9,10-epoxy-12,15-octadecadienoate	C19H32O3	LMFA01070012	7.41	309.24	1.79	0.005736	↑	2.057
4	Oleoyl glycine	C20H37NO3	HMDB0013631	5.09	381.31	1.66	0.00000792	↑	2.099
5	Dextrorphan O-glucuronide	C23H31NO7	HMDB0010341	3.01	398.19	1.50	0.002671	↑	2.473
6	Butyl (S)-3-hydroxybutyrate glucoside	C14H26O8	HMDB0031694	6.27	303.14	2.02	0.00000238	↓	0.393
7	(R)-1-O-[b-D-Apiofuranosyl-(1->2)-b-D-glucopyranoside]-1,3-octanediol	C22H40O9	HMDB0032798	5.77	473.21	1.58	0.001802	↓	0.466
8	2-Amino-5-phenylpyridine	C12H10N2	HMDB0029747	3.10	212.11	1.70	0.00005654	↓	0.419
9	Cer (t18:0/16:0(2OH))	C34H69NO5	LMSP02030015	10.80	572.52	2.24	0.00009182	↓	0.386
10	Janthitrem G	C39H51NO6	HMDB0030531	6.65	612.37	2.20	0.00007879	↓	0.451
11	PI (20:0/20:4(5Z,8Z,11Z,14Z))	C49H87O13P	HMDB0009869	6.53	469.29	2.40	0.0000000019	↓	0.428
12	Ganglioside GA2	C56H102N2O18	HMDB0004889	6.48	557.35	2.17	0.004054	↓	0.404
13	13-Hydroxy-5′-O-methylmelledonal	C24H30O9	HMDB0035864	5.10	507.15	1.53	0.003510	↓	0.453

Note: formula: chemical formula of metabolite; library ID: accession number of metabolites in the corresponding database; HMDB: HMDB database number; LMSP and LMFA: lipid MAP database number; retention time: refers to the retention time of charged ions in the chromatogram; *M*/*Z*: mass charge ratio, refers to the ratio of the mass of charged ion to the charge charged; VIP: VIP value of this metabolite in OPLS-DA model between the two groups; *P*: the result of significance test of difference of this metabolite between the two groups; Exp: indicates that the expression level of the substance is upregulated in cancer tissues; indicates that the expression level of the substance is downregulated in cancer tissues; FC: the fold change of differential expression of this metabolite between the two groups; as well as CA/NO.

## Data Availability

The datasets used and/or analyzed during the current study are available from the corresponding author on reasonable request.

## Supplementary Materials

Supplementary Materials Supplementary Table 1: HMDB hierarchical classification and number of metabolisms.
