# Supplementary material for: Metabolomics of Esophageal Squamous Cell Carcinoma Tissues: Potential Biomarkers for Diagnosis and Promising Targets for Therapy
Source: Biomed Res Int. 2022 Jun 23;2022:7819235. doi: 10.1155/2022/7819235 (PMC9246618; doi:10.1155/2022/7819235)
Supplement: Supplementary Materials — Supplementary Table 1: HMDB hierarchical classification and number of metabolisms. [file 7819235.f1.docx]

| Supplementary table1 HMDB hierarchical classification and number of metabolisms | | |
| --- | --- | --- |
| Hierarchical levels | Number | Percent(%) |
| Superclass |  |  |
| Lipids and lipid-like molecules | 426 | 62.63 |
| Organic acids and derivatives | 82 | 11.82 |
| Organoheterocyclic compounds | 43 | 6.28 |
| Organic oxygen compounds | 41 | 5.99 |
| Phenylpropanoids and polyketides | 40 | 5.84 |
| Benzenoids | 18 | 2.63 |
| Organic nitrogen compounds | 13 | 1.9 |
| Alkaloids and derivatives | 9 | 1.31 |
| Nucleosides, nucleotides, and analogues | 8 | 1.17 |
| Hydrocarbons | 2 | 0.29 |
| Other | 1 | 0.15 |
|  |  |  |
| Class |  |  |
| Fatty Acyls | 146 | 21.31 |
| Glycerophospholipids | 129 | 18.83 |
| Prenol lipids | 79 | 11.53 |
| Carboxylic acids and derivatives | 70 | 10.22 |
| Organooxygen compounds | 41 | 5.99 |
| Steroids and steroid derivatives | 39 | 5.69 |
| Sphingolipids | 23 | 3.36 |
| Organonitrogen compounds | 13 | 1.9 |
| Glycerolipids | 13 | 1.9 |
| Benzene and substituted derivatives | 12 | 1.75 |
| Other | 120 | 17.52 |
|  |  |  |
| Subclass |  |  |
| Aminoacids,peptides,and analogues | 66 | 9.64 |
| Glycerophosphoethanolamines | 56 | 8.18 |
| Fatty acids and conjugates | 52 | 7.59 |
| Glycerophosphocholines | 47 | 6.86 |
| Not Available | 42 | 6.13 |
| Fatty alcohols | 22 | 3.21 |
| Fatty acid esters | 21 | 3.07 |
| Carbohydrates and carbohydrate conjugates | 21 | 3.07 |
| Lineolic acids and derivatives | 19 | 2.77 |
| Eicosanoids | 19 | 2.77 |
| Other | 320 | 46.72 |

Note: According to the number of metabolites, the name of the selected HMDB level (Superclass, Class or Subclass) and the percentage of metabolites are displayed in order from high to low.
